# Morphological Response of Eight *Quercus* Species to Simulated Wind Load

**DOI:** 10.1371/journal.pone.0163613

**Published:** 2016-09-23

**Authors:** Tonggui Wu, Peng Zhang, Lei Zhang, Geoff G. Wang, Mukui Yu

**Affiliations:** 1 East China Coastal Forest Ecosystem Long-term Research Station, Research Institute of Subtropical Forestry, Chinese Academy of Forestry, Hangzhou, Zhejiang 311400, PR China; 2 Department of Forestry and Environment Conservation, Clemson University, Clemson, SC29634-0317, United States of America; Centro de Investigacion Cientifica y de Educacion Superior de Ensenada Division de Fisica Aplicada, MEXICO

## Abstract

Leaf shape, including leaf size, leaf dissection index (LDI), and venation distribution, strongly impacts leaf physiology and the forces of momentum exerted on leaves or the canopy under windy conditions. Yet, little has been known about how leaf shape affects the morphological response of trees to wind load. We studied eight *Quercus* species, with different leaf shapes, to determine the morphological response to simulated wind load. *Quercus* trees with long elliptical leaves, were significantly affected by wind load (*P*< 0.05), as indicted by smaller specific leaf area (SLA), stem base diameter and stem height under windy conditions when compared to the control. The *Quercus* trees with leaves characterized by lanceolate or sinuous edges, showed positive morphological responses to wind load, such as bigger leaf thickness, larger stem diameter, allocation to root biomass, and smaller stem height (*P*< 0.05). These morphological responses to wind can reduce drag and increase the mechanical strength of the tree. Leaf dissection index (LDI), an important index of leaf shape, was correlated with morphological response to wind load (*P*< 0.05), including differences in SLA, in stem base diameter and in allocation to root biomass. These results suggest that trees with higher LDI, such as those with more and/or deeper lobes, are better adapted to wind load.

## Introduction

Thigmomorphogenesis is the response of plants to mechanical sensation, such as wind or raindrops, by altering their growth patterns [1–3]. It generally results in common morphological variation among plants without phylogenesis [4–5]. For example, reduced stature and increased thickening of the stem can prevent stem failure by reducing aerodynamic drag or by increasing mechanical strength [6–8]. Trees are subject to greater impacts from wind load than other plants due to their tall stature [9]. To survive, trees may develop a “stunted” appearance under wind load, which can decrease the speed-specific drag of the crown [7, 10]. As a response, leaf size and area, and stem height decreases [11–14], whereas leaf thickness, stem diameter, and root-to-shoot ratio increases [6, 15–16]. Morphological responses to wind were also found to vary among species within same genus, and even within species, although they have similar ecophysiology and morphology [4, 17–20].

Leaves tend to be vibrated, deformed and reconfigured under wind load [21–22]. The shape of the leaf, such as leaf size, leaf dissection index (LDI), and venation distribution, could regulate momentum forces on leaves and woody portions as a whole on the canopy. These forces can indirectly influence the leaf physiology under wind load [23–24], due to a close correlation between leaf shape and physiology [25–26]. Vogel [21] suggested that leaves with lobed bases had lower drag and fluttered less than leaves with acute bases. Therefore, leaf shape may play an important function on plant response to wind load, for example, leaf tooth size may be linked to wind speed for *Quercus kelloggii* [27], but how leaf shape impacts the response and adaptation to wind for trees has not been well understood.

**Fig 1:**
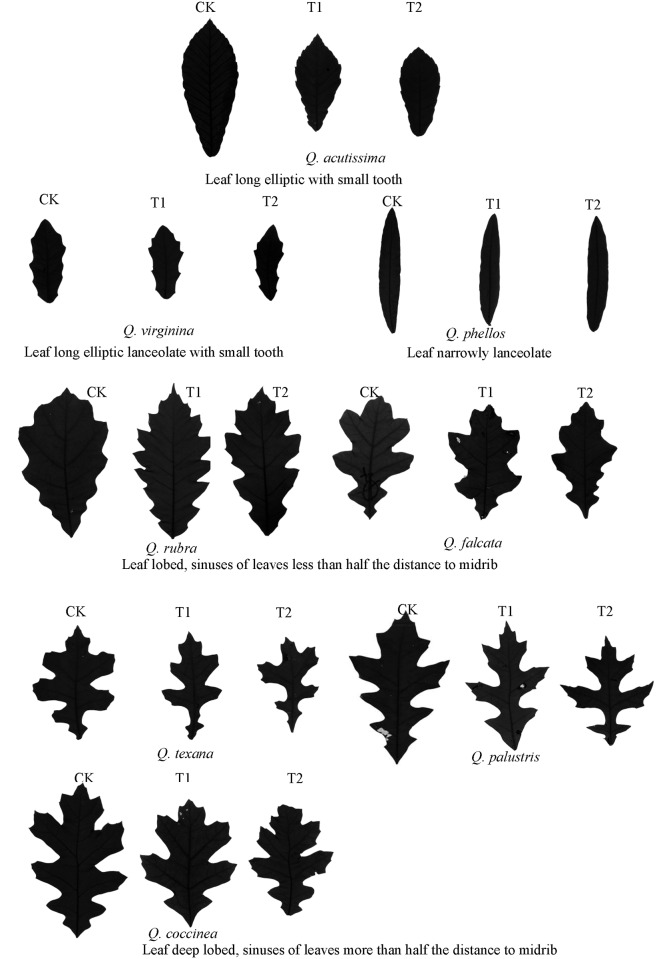
Leaves of eight *Quercus* species under simulated wind load.

The genus *Quercus* is distributed widely in the Northern Hemisphere, especially in Asia and the Americas. It has deciduous and evergreen types with diverse leaf shapes, including lanceolate and oval, and serrated and entire leaf margins [28], which makes it an ideal genus for studying the function of leaf shape under wind load. We selected eight *Quercus* species with different leaf shapes (see Fig 1), which are common in coastal windy areas of the Northern Hemisphere, to determine their physiological (photosynthesis, transpiration), and morphological (including leaf, stem, root and biomass) response to wind load. In a previous study, physiological responses of the eight species were found to differ under wind load [29]. In this study, we further investigate1) how *Quercus* species with different leaf shapes respond to wind load in morphology, and 2) the relationship between leaf shape and the responses of morphology for *Quercus* species under wind load.

## Materials and Methods

### Materials and growing conditions

**Fig 2:**
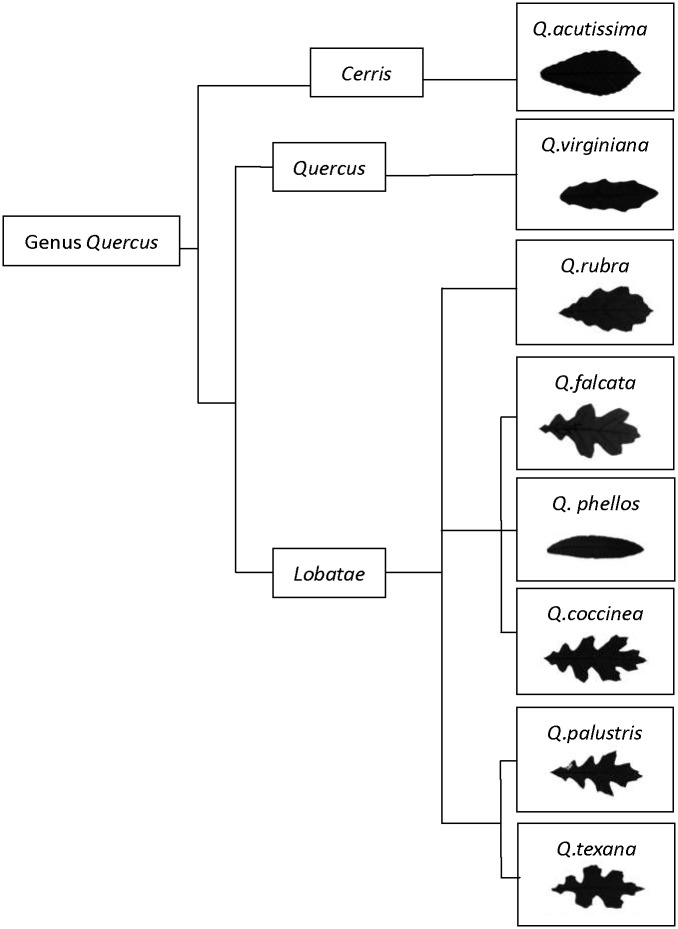
Phylogenetic relationships among eight *Quercus* species.

Eight *Quercus* species (see Figs 1 and 2), two white oaks (*Q*. *acutissima* and *Q*. *virginiana*), and six red oaks (*Q*. *phellos*, *Q*. *rubra*, *Q*. *falcata*, *Q*. *texana*, *Q*. *palustris*, and *Q*. *coccinea*) were selected due to large differences in leaf shape for this study. Based on the methods of Willan [30], seeds of *Quercus* were collected from 15–20 seed trees, chosen from one natural forest stand over 30 years old for each species in 2011. Selected trees were only those that had grown above the average canopy, had straight form, and were free from disease and pests. Trees sampled from the same stand were at least 50 m apart from each other. Seeds were then sown in the nursery at Research Institute of Subtropical Forestry in Hangzhou, China, in 2012. Seedlings, 100 individuals for each species, were transplanted to pots 20 cm in diameter and 25 cm deep in January 2013. All transplanted seedlings were acclimated for one month in a greenhouse with air temperature between 20 and 35°C. Fifty-four average size seedlings per species were then selected for the study.

### Experimental design

Nine rooms were constructed from glass with a size of 2 m × 2 m ×2 m and were housed within a greenhouse. Three treatments were designed: control (CK), about 4 m s^-1^ wind speed (T1) and about 6 m s^-1^ wind speed (T2). Each treatment had three replicates, and they were randomly assigned to each of the nine rooms. In each room, eight *Quercus* species, with six seedlings of each species, were randomly placed in a row. Wind load was produced by electric-powered fans for two one-hour durations at 0:00 and 12:00 from the 1^st^ March to 7^th^ October, following the procedure developed by Murren and Pigliucci [19]. Each day, each species was moved one row from left to right, and individual trees were moved within the row, to ensure that each species and individual were subjected to similar wind exposure in each treatment room [13]. All treatments were identical except for wind load. The trees were watered daily with tap water to compensate for evaporative loss.

### Leaf morphology, stem growth, and biomass measurements

Stem height and base diameter were determined with a ruler and vernier calipers before and after the experiment. The growth of the stem was defined as the increase in height (or increment) during the experiment period.

After the experiment, healthy mature leaves were sampled for determining leaf morphology. Thirty leaves were selected from six plants of each tree species from each treatment room, and scanned to produce digitized images. Leaf length, width, perimeter, and area were analyzed by Wseen Leaf Area Analysis Systems (Wseen Co. Ltd, China), and subsequently oven dried at 60°C to a constant dry weight, and then weighed to the nearest 0.001 g using an electronic balance (JA12002, Jinghai Instruments Co., Ltd., Shanghai, China). Specific leaf area (SLA) was calculated as leaf area/mass. Leaf dissection index (LDI), an important index of leaf shape, was calculated by leaf perimeter/square root of leaf area [26]. The more and deeper leaf serrations are, the higher the LDI value is. A portion of twenty leaves sampled, with size of 0.5 cm ×1.0 cm, from six plants of each tree species per treatment room were fixed with Formalin-acetic acid-alcohol (FAA), and leaf anatomy, such as leaf thickness and leaf vein thickness, were determined by digital microscope (Motic B5 Professional Series, Bock Optronics Inc., Canada) and Motic Images Advanced 3.0 (Micro-Optic Industrial Group Co., Ltd., China).

After the experiment, all seedlings were harvested. Leaf, shoot, stem, and roots of each seedling were destructively sampled for each tree species in each room, and oven dried for their constant weight at 60°C, then biomass for each organ and total biomass were weighed and determined.

### Stem mechanical property

Each stem was cut to bend on a tangential plane with simple beams supporting each end of the stem, the distance between the two supported ends was 120 mm, and was referred to as the span length (L). Diameter (D) of the stem center sections was also recorded. The stem was displaced at 0.5 mm s^-1^, and the maximum bending force (F) was determined using universal testing machine (RGE-2100, Shenzhen Reger Inc., China) [4, 31].

Stem bending strength (∂) is a material property, defined as the stress in a material just before it yields in a flexure test [32]. It was calculated by maximum bending moment (M) and section modulus (W).

∂=M/W (1)

M=F×L/2 (2)

$$W=\pi \times D^{3}/32$$ (3)

Where ∂ is bending strength (kN mm^-2^), D is stem diameter (mm), F is the load (force) at the fracture point (N), and L is span length (L = 120 mm in this study).

### Statistical analysis

Mean values of each index for each tree species by room unit were used for statistical analysis. The responses of trees to wind load in leaf morphology, stem growth and biomass allocation were expressed by the differences in SLA, in stem diameter and in percentage of root biomass between T2 and CK, which produced significant effects on trees, respectively. The differences (D) were determined using the following equation [9]:

$$D=(T_{2}-CK)/CK\times 100\%$$ (4)

WhereT_2_ is the variable for trees under T2 treatment, CK is the variable for trees in the control.

General linear model (GLM) was applied to separate the variance explained by species, treatment, the interaction between them, and random effect of room. The difference among wind treatments were then analyzed by one-way ANOVA. Post-hoc statistical groupings were determined with a stringent Bonferroni correction. Simple linear regression was used to test relationships between LDI under control and the differences in morphology (the differences in SLA, in stem diameter, and in percentage of root biomass under T2) for the eight tree species. All analyses were performed with SPSS software package version 15.0 (SPSS, Chicago, IL).

## Results

### Leaf morphological response to simulated wind load

Leaf morphology showed significant differences among tree species, and leaf length, leaf thickness, vein thickness and specific leaf area (SLA) also showed different responses to simulated wind load for eight *Quercus* species (S1 Table). SLA of all tree species under T2 was significantly smaller than that under CK (Table 1). Leaf length was also significantly shorter by 12.43%– 17.56% for *Q*. *acutissima*, *Q*. *palustirs* and *Q*. *coccinea*, while leaf thickness or vein thickness was significantly bigger by 8.40%– 31.85% for *Q*. *virginiana*, *Q*. *phellos*, *Q*. *rubra*, *Q*. *texana*, *Q*. *palustris* and *Q*. *coccinea* under T2 than those under CK (Table 1).

**Table 1:** Leaf morphology (means ±SE) of eight *Quercus* species under simulated wind load (n = 3).

Species	Treatment	Length cm	Width cm	Thickness μm	Vein thickness μm	SLA cm^2^ g^-1^	LDI
***Q*. *acutissima***	**CK**	**11.50±1.01 A**	3.96±0.59	128.96±10.32	807.83±168.97	**41.83±4.47 A**	5.34±0.26
	**T1**	**10.18±0.92 B**	3.59±0.35	123.05±5.69	877.55±179.46	**50.38±9.27 A**	5.29±0.28
	**T2**	**10.07±1.28 B**	3.49±0.37	126.39±9.96	838.42±62.46	**35.87±2.46 B**	5.71±0.50
***Q*. *virginiana***	**CK**	6.26±0.39	2.67±0.31	**135.24±3.80 B**	**598.45±129.11 B**	**34.16±3.23 A**	5.23±0.34
	**T1**	6.26±0.62	2.68±0.39	**162.53±23.57 A**	**584.29±66.17 B**	**37.65±5.16 A**	5.18±0.20
	**T2**	6.19±0.51	2.63±0.30	**160.38±29.72 A**	**723.30±83.52 A**	**29.03±1.37 B**	5.43±0.60
***Q*. *phellos***	**CK**	8.59±1.05	1.43±0.28	**115.44±28.23 B**	635.64±123.65	**50.39±5.10 A**	6.27±0.29
	**T1**	7.88±0.74	1.41±0.15	**114.07±6.58 B**	566.85±114.89	**41.21±6.18 B**	6.29±0.43
	**T2**	8.93±0.45	1.40±0.14	**152.21±25.09 A**	673.03±52.71	**40.49±2.36 B**	6.84±0.32
***Q*. *rubra***	**CK**	11.91±1.33	7.75±1.12	125.21±8.44	**722.00±135.07 C**	**106.82±8.83 A**	6.95±0.58
	**T1**	13.20±1.01	8.17±0.97	126.21±6.33	**1040.20±121.52 A**	**115.57±8.39 A**	6.54±0.32
	**T2**	11.98±1.36	7.74±0.66	135.24±8.77	**911.56±30.07 B**	**93.93±7.41 B**	7.25±0.33
***Q*. *falcata***	**CK**	10.61±0.31	6.62±0.17	125.36±12.49	955.88±184.10	**85.71±5.90 A**	6.23±0.53
	**T1**	10.77±1.26	6.79±0.62	121.62±14.21	880.67±228.59	**77.13±6.86 B**	6.04±0.33
	**T2**	10.72±0.85	6.70±0.56	120.15±7.00	964.96±192.92	**76.09±6.81 B**	6.84±0.84
***Q*. *texana***	**CK**	9.28±1.00	5.40±1.10	137.00±7.49	**646.65±124.22 B**	**90.93±10.61 A**	7.80±0.48
	**T1**	9.54±0.95	5.020±0.62	126.26±18.97	**723.31±75.61 AB**	**94.97±5.42 A**	8.10±0.86
	**T2**	8.46±0.96	4.82±0.59	133.69±8.46	**821.70±137.89 A**	**66.93±5.52 B**	8.88±2.09
***Q*. *palustris***	**CK**	**12.12±0.99 A**	6.67±1.22	127.79±10.93	**750.15±58.59 B**	**106.98±8.19 A**	8.05±0.31
	**T1**	**10.21±1.05 B**	6.20±0.65	129.72±15.40	**870.74±103.16 A**	**114.45±9.37 A**	8.07±0.47
	**T2**	**10.31±0.42 B**	6.48±0.70	136.30±11.65	**813.04±94.51 AB**	**84.75±6.66 B**	8.22±0.55
***Q*. *coccinea***	**CK**	**12.23±1.26 A**	7.27±1.16	119.38±10.73	**760.83±124.02 B**	**52.72±5.61 A**	7.55±0.47
	**T1**	**11.04±1.54 AB**	7.01±0.96	115.86±8.95	**930.91±199.55 A**	**46.67±5.52 AB**	6.42±0.71
	**T2**	**10.12±1.34 B**	7.62±1.22	128.74±26.21	**947.70±146.71 A**	**38.74±3.98 B**	8.22±1.15

SLA, specific leaf area; LDI, leaf dissection index. CK, control; T1, about 4 m s^-1^ wind speed, and T2, about 6 m s^-1^ wind speed. The different letters in the same column meant significant difference at 0.017 (after Bonferroni correction) level.

### Stem growth response to simulated wind load

**Fig 3:**
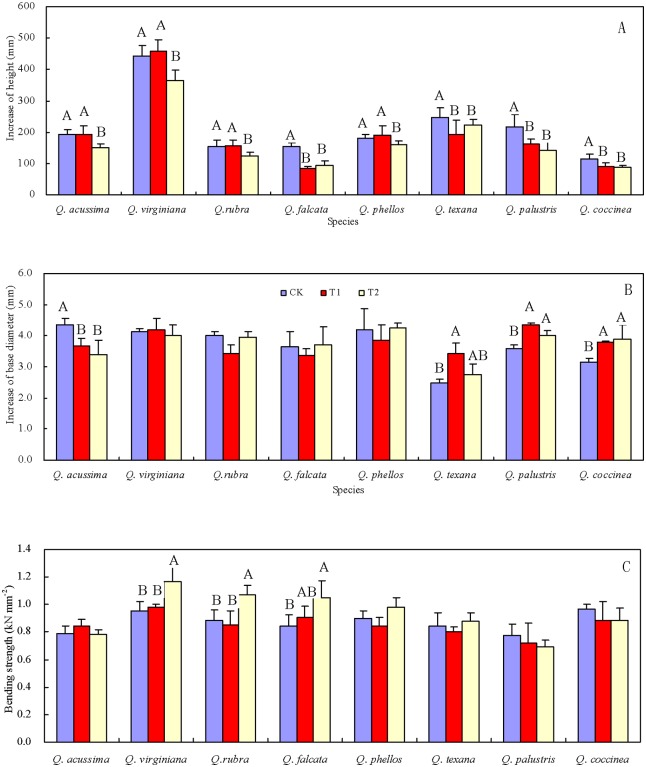
The stem traits (means + SE) for eight *Quercus* species under simulated wind load. (A) growth of stem height, (B) base diameter, (C) stem bending strength. The different letters in the same column meant significant difference at 0.017 (after Bonferroni correction) level (n = 3).

The growth of stem height under T1was significantly higher for *Q*. *falcta*, *Q*. *texana*, *Q*. *palustris* and *Q*. *coccinea* than that under CK, and it is same under T2 for all species (Fig 3A, S2 Table). The growth of stem base diameter under T1 and T2 was also thinner for *Q*. *acutissima* than that under CK, but was significant larger for *Q*. *texana*, *Q*. *palustris* and *Q*. *coccinea* (Fig 3B, S2 Table). Stem bending strength under T2 was bigger for *Q*. *virginiana*, *Q*. *rubra* and *Q*. *falcate* than that under CK (Fig 3C, S2 Table).

### Biomass allocation response to simulated wind load

Total biomass was smaller for *Q*. *acutissima*, *Q*. *rubra* and *Q*. *falcata* under wind load than that under CK (Table 2). Percentage of root biomass allocation was bigger and the leaf biomass allocation was smaller under T2 than those under CK for *Q*. *acutissima* and *Q*. *virginiana* (Table 2). Percentage of root biomass was bigger and the stem allocation was smaller under T2 than those under CK for *Q*. *rubra*, *Q*. *falcata* and *Q*. *phellos*. Biomass allocation to each organ was insignificantly influenced by wind load for *Q*. *texana*, *Q*. *palustris* and *Q*. *coccinea*.

**Table 2:** Biomass and percentages of biomass allocation (means ±SE) for eight *Quercus* species under simulated wind load (n = 3).

Species	Treatment	Total g	Percentages of biomass allocation		
			Leaf %	Stem %	Root %
***Q*. *acutissima***	**CK**	**65.02±5.88 A**	**25.89±1.94 A**	33.82±2.86	**40.28±3.00 B**
	**T1**	**55.74±4.96 B**	**19.83±1.85 B**	32.04±5.94	**48.12±2.35 B**
	**T2**	**54.83±4.31 B**	**14.68±2.02 B**	29.30±6.11	**56.01±3.88 A**
***Q*. *virginiana***	**CK**	24.33±2.55	**35.11±4.15 A**	30.53±3.37	**34.36±2.14 B**
	**T1**	23.20±2.39	**30.62±2.98 AB**	29.90±3.15	**39.48±2.80 AB**
	**T2**	23.30±2.79	**25.86±3.56 B**	27.47±3.52	**46.68±3.48 A**
***Q*. *phellos***	**CK**	46.46±2.15	21.23±4.15	**32.35±5.48 A**	**46.43±5.55 B**
	**T1**	41.69±3.56	18.24±1.46	**29.69±5.73 AB**	**52.07±9.08 A**
	**T2**	40.55±3.18	18.78±4.02	**26.75±4.33 B**	**54.46±5.74 A**
***Q*. *rubra***	**CK**	**74.03±4.32 A**	13.28±2.99	**25.85±4.82 A**	**60.86±2.93 B**
	**T1**	**70.10±5.99 AB**	13.84±4.08	**26.23±7.40 A**	**59.93±5.61 B**
	**T2**	**67.43±3.17 B**	14.12±1.97	**20.83±5.73 B**	**65.05±3.39 A**
***Q*. *falcata***	**CK**	**68.73±3.17 A**	14.94±3.18	**41.63±3.74 A**	**43.43±4.68 B**
	**T1**	**60.80±4.26 B**	14.64±3.01	**38.10±3.93 AB**	**47.27±5.85 AB**
	**T2**	**60.84±3.15 B**	13.78±3.85	**33.65±2.71 B**	**52.57±6.88 A**
***Q*. *texana***	**CK**	57.45±4.18	15.50±2.47	40.52±3.74	43.97±3.86
	**T1**	58.76±5.66	16.63±4.39	38.02±6.19	45.35±4.03
	**T2**	55.03±5.84	14.26±3.61	40.83±5.78	44.91±5.67
***Q*. *palustris***	**CK**	60.91±5.33	15.24±1.89	31.71±2.33	53.05±7.49
	**T1**	57.13±3.69	14.32±1.51	29.53±3.76	56.16±10.22
	**T2**	58.22±5.84	13.81±2.28	29.87±4.00	56.32±7.16
***Q*. *coccinea***	**CK**	31.87±3.32	27.31±7.01	22.57±4.87	50.12±7.65
	**T1**	30.09±5.69	25.21±6.38	21.88±5.42	52.91±8.18
	**T2**	30.07±4.86	25.46±4.72	21.76±6.22	52.78±10.48

The different letters in the same column meant significant difference at 0.017 (after Bonferroni correction) level.

### Relationship between leaf shape and morphological response to wind load

**Fig 4:**
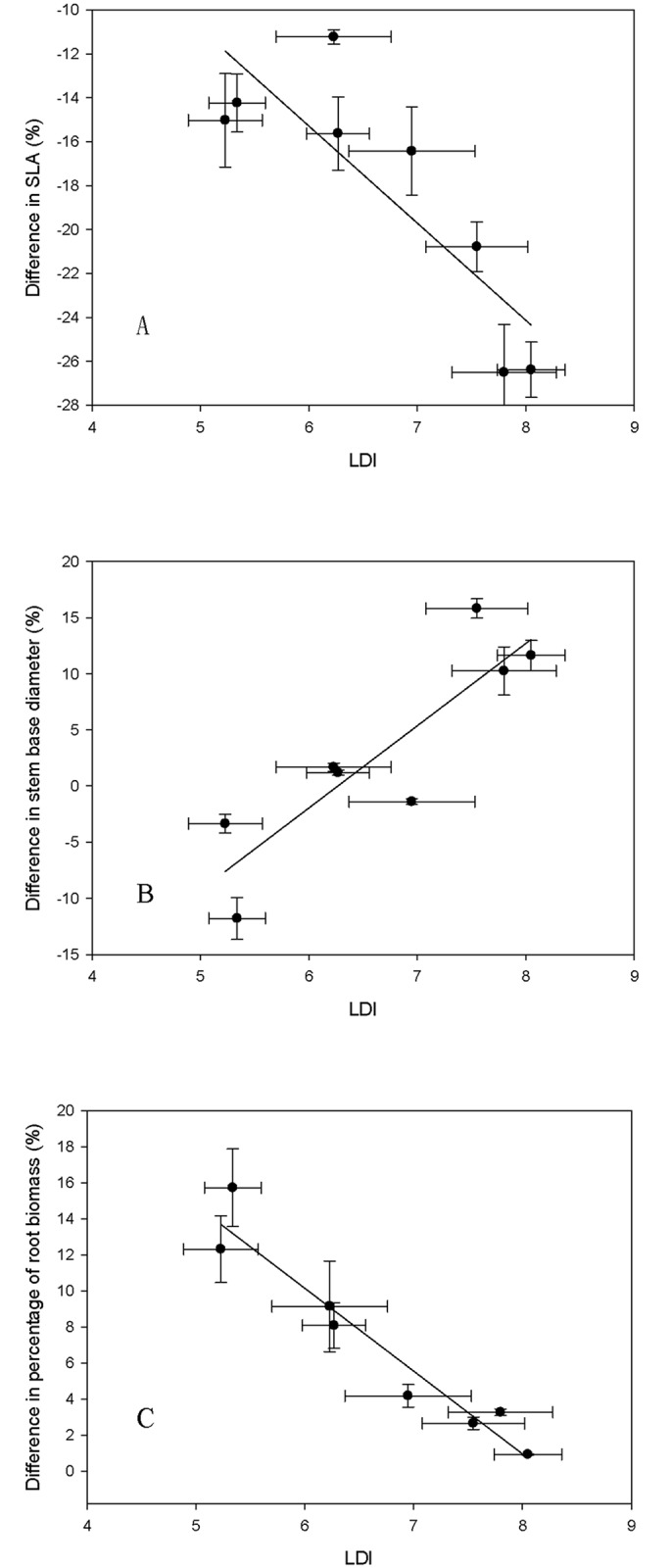
Regressions between leaf dissection index (means + SE) and the differences in morphological index (means + SE). Linear regressions (A) between LDI and difference in SLA (*y* = -4.424 *x*+ 11.267, *R*^2^ = 0.710), (B) between LDI and difference in stem diameter (*y* = 7.323 *x*- 45.900, *R*^2^ = 0.768), and (C) between LDI and difference in percentage of root biomass (*y* = -4.596 *x*+ 37.723, *R*^2^ = 0.927).

Leaf dissection index (LDI) showed negative correlations with differences in SLA and percentage of root biomass (*R*^2^ = 0.710, *P* = 0.009; *R*^2^ = 0.927, *P* = 0.000, respectively) (Fig 4A and 4C, S3 Table), and showed a positive correlation with stem diameter (*R*^2^ = 0.768, *P* = 0.004) (Fig 4B, S3 Table).

## Discussion

### Morphological responses of eight *Quercus* species to wind load

Plants show various responses to mechanical sensation. Jaffe [1] defined the term “thigmomorphogenesis” to describe the physiological, biochemical and morphological responses of plants to wind and other mechanical perturbations. Although previous studies showed the responses to wind are different than pure mechanical perturbation, dynamic perturbation, such as simulated wind load, was more accurate than static perturbation resulting in effective mechanical signals produced by leaf or branch movements, which can bring a series of physiological, biochemical and morphological responses [33].

The drag of a tree, induced by wind, is mainly determined by the exposed surface area. The exposed area of broad-leaved tree are its leaves, which produce wind resistance that can provoke bending, breakage, and up-rooting [34–35]. Under windy conditions, a reduction in leaf area would reduce breakage of branches [6, 14], especially in broad-leaved species. Similarly, leaves or their veins would thicken to support and protect leaves from damage [16, 36–37], which was supported by the observed response in leaf or leaf vein thickness for *Q*. *virginiana*, *Q*. *phellos*, *Q*. *rubra*, *Q*. *texana*, *Q*. *palustris* and *Q*. *coccinea*. SLA would decrease to reduce drag and increase toughness due to a decrease in leaf area and an increase in leaf thickness under wind [38], and this is confirmed by our result: SLA was significantly smaller under wind load for all trees. Meanwhile, higher leaf vein density and thickness are known to contribute to higher maximum leaf hydraulic conductance [6, 18, 39]. Leaf transpiration rates increased under windy conditions for all *Quercus* species, except *Q*. *acutissima*, in a previous study [29].

In many plants, reduced stem elongation and increased stem diameter were reported as responses to wind load [40]. Bending exerted by a given wind force scales linearly with plant height [9]. Trees decrease in height with increasing wind load, resulting in a “stunted” appearance [10]. Trees respond by increasing the amount of secondary wood with high microfibrillar angles and spiral grain, and by producing thicker trunks and roots [13, 15, 41–42]. These adaptive strategies help trees reduce wind load by reducing the amount of drag. Similarly, in this study, it was found that all species had lower growth in stem height, and *Q*. *texana*, *Q*. *palustris* and *Q*. *coccinea* had higher growth in stem diameter in response to wind load. Stem mechanical strength also increases for plants as a mechanism to protect them from breaking under windy conditions [4], and in this study, bending strength under wind load was higher for *Q*. *virginiana*, *Q*. *rubra* and *Q*. *falcata* than that under the control (*P*< 0.05, Fig 3c). These findings are consistent with Coutand’s hypothesis: plants may acclimate to the imposed strain [33]. For *Q*. *acutissima*, both the growth of stem height and diameter were smaller under wind load, indicating that its growth was seriously restricted under wind load [6]. There may be a threshold of wind stress for each species, and trees may not be perceived or be affected below this threshold [43]. This is why different responses to T1 and T2 were found for some species.

Biomass is allocated to the organs that are less affected by wind load, like roots [6, 33]. Allocation of biomass to roots is advantageous because it increases the magnitude of the mechanical forces required to uproot a plant from its substrate. In our study, the response of biomass allocation to wind load varied with leaf shape (Table 2). In addition, although leaf photosynthesis decreased for all species [29], the difference in percentage of root biomass was largerby15.72% for *Q*. *acutissima*, 12.31% for *Q*. *virginiana*, and around 4%– 9% for *Q*. *rubra*, *Q*. *falcata* and *Q*. *phellos* under T2 than those under the control. However, the biomass allocation to each organ was stable for *Q*. *texana*, *Q*. *palustris* and *Q*. *coccinea* under wind load (Table 2), indicating that these tree species are less influenced by wind.

Phylogenetic relationships among the eight *Quercus* species are shown in Fig 2 based on DNA sequences analysis reported in Hubert et al [44]. *Q*. *acutissima* from the group *cerris*, and *Q*. *virginiana* from the group *Quercus*, had different morphological responses to wind than species from group *Lobatae*. Species with close phylogenetic relationships, such as *Q*. *texana* and *Q*. *palustris*, had similar morphological responses to wind. However, *Q*. *coccinea*, which is more phylogentically related to *Q*. *phellos* and *Q*. *falcata*, showed a similar morphological response to *Q*. *texana* and *Q*. *palustris*.

### Effect of leaf shape on morphological responses to wind load

Here, leaf shape and morphology showed significantly differences for eight *Quercus* species, which provided good materials for studying the function of leaf shape under wind load (S1 Table). Both *Q*. *acutissima*, with long elliptic leaf, and *Q*. *virginiana*, with long elliptic lanceolate leaf, exhibited lower LDI than other species. While, *Quercus* with leaf lobed, showed higher LDI, and the LDI increased with the depth of lobe (Table 1 and Fig 2).

Under windy conditions, the drag on leaves will be larger than the force imposed on the trunk and branches [24]. Leaf shape, including edge characteristics, and surface smoothness, may affect the air flow over the leaf. These characteristics regulate the force caused by wind on the leaves and woody portions of the entire canopy, which can further influence the leaf physiology [27, 45–47]. Wind flows more easily through needles or lanceolate leaves than broad-leaved species due to their smaller area, greater flexibility, and tight clustering in high wind [48]. Meanwhile, lobed leaves often have a slight upward curl to decrease drag from extreme winds, and they curl upward resulting in the upper leaf surface forming the core of a cone [24]. In previous studies, leaves of *Q*. *kelloggii* [27] and *Sasssafras albidum* [49] where found to have bigger and more numerous lobes in windy areas. Therefore, trees with lanceolate and lobed leaves may have lower drag and be less effected than broad-leaved trees under similar wind speeds. This indicates why leaf morphology, stem growth and biomass allocation were less impacted by wind for *Quercus* species with lanceolate and lobed leaves in this study.

The value of LDI increases with increasing leaf dissection, for example, leaves with more and/or deeper lobes would have a larger LDI [50]. In this study, the difference in SLA decreased and stem diameter increased with increasing LDI, suggesting that the adaptation of *Quercus* trees to wind load would manifest in leaf shape. Trees with leaves that with more and deeper lobes (toothed) were better adapted to wind load, having decreased leaf area and increased stem diameter, with no influence on biomass allocation. Trees with elliptical leaves without lobes however, had restricted growth under windy conditions, and allocated more biomass to roots. Therefore, LDI showed negative correlation with the percentage of root biomass. As a result, lobed or serrated leaves are not easily damaged in windy conditions due to fluid mechanics [24, 51].

## Conclusion

Among the eight *Quercus* species, *Q*. *acutissima*, with leaves characterized by long elliptic and small-toothed edges, was significantly affected by wind load, as indicted by a decrease in leaf area and stem growth. The tree species, that are characterized by leaves that are lanceolate or with sinuous edges, showed wind-adapted morphological responses to wind load, such as increasing leaf thickness, stem diameter, biomass allocation to roots, and decreasing stem height. Leaf dissection index (LDI) was correlated with morphological responses. Trees with higher LDI, such as those with more and/or deeper lobes, being better adapted to wind load. This study also suggested that the biomechanical response of trees would lead us to better understanding tree adaptation to wind load. An intensive study on biomechanical response of trees to wind would be significant for species selection in the establishment of shelterbelt system in windy areas.

## Supporting Information

S1 Table Effects (mean square) of species, wind treatments and their interaction on leaf morphology. Significance levels: *** = p < 0.001; ** = p < 0.01; * = p < 0.05.
(DOCX)

S2 Table The increase of base diameter and height, and stem bending strength for eight *Quercus* species under simulated wind load. CK, control; T1, about 4 m s^-1^ wind speed, and T2, about 6 m s^-1^ wind speed.
(DOC)

S3 Table LDI of eight species under CK and differences in SLA, stem base diameter and percentage of root biomass for eight species. SLA, specific leaf area; LDI, leaf dissection index.
(DOC)
