# Supplementary material for: Morphological Response of Eight Quercus Species to Simulated Wind Load
Source: PLoS One. 2016 Sep 23;11(9):e0163613. doi: 10.1371/journal.pone.0163613 (PMC5035066; doi:10.1371/journal.pone.0163613)
Supplement: S1 Table — Significance levels: *** = p < 0.001; ** = p < 0.01; * = p < 0.05. (DOCX) [file pone.0163613.s001.docx]

|  | | Species | Wind | Species × Wind | Error |
| --- | --- | --- | --- | --- | --- |
| Length | | 648.32^***^ | 168.63^**^ | 226.55^**^ | 93.17 |
| Width | | 225.69^***^ | 15.37 | 96.11^**^ | 118.19 |
| Thickness | | 8542.64^***^ | 996.73^**^ | 3211.18^**^ | 1106.33 |
| Vein thickness | | 55371.26^***^ | 12867.59^**^ | 16591.77^**^ | 8674.32 |
| SLA | | 2671.58^***^ | 966.31^**^ | 1367.29^**^ | 619.38 |
| LDI | | 366.42^***^ | 53.16 | 96.27^*^ | 157.83 |
|  |  |  |  |  |  |
