# Supplementary material for: Morphological Response of Eight Quercus Species to Simulated Wind Load
Source: PLoS One. 2016 Sep 23;11(9):e0163613. doi: 10.1371/journal.pone.0163613 (PMC5035066; doi:10.1371/journal.pone.0163613)
Supplement: S2 Table — CK, control; T1, about 4 m s-1 wind speed, and T2, about 6 m s-1 wind speed. (DOC) [file pone.0163613.s002.doc]

| Species | Treatment | Increase of base diameter  mm | Increase of height  mm | Bending strength  kN mm^-2^ |
| --- | --- | --- | --- | --- |
| *Q. acutissima* | CK | 4.33±0.24 | 193.33±16.07 | 0.79±0.05 |
|  | T1 | 3.67±0.26 | 193.78±27.30 | 0.84±0.05 |
|  | T2 | 3.39±0.47 | 150.00±13.64 | 0.78±0.04 |
| *Q. virginiana* | CK | 4.14±0.09 | 443.33±32.96 | 0.95±0.07 |
|  | T1 | 4.20±0.35 | 458.06±35.56 | 0.98±0.03 |
|  | T2 | 4.00±0.35 | 363.80±34.36 | 1.16±0.13 |
| *Q. phellos* | CK | 4.20±0.67 | 180.28±13.00 | 0.90±0.05 |
|  | T1 | 3.86±0.50 | 190.80±30.05 | 0.84±0.06 |
|  | T2 | 4.25±0.17 | 159.72±13.40 | 0.98±0.07 |
| *Q. rubra* | CK | 4.00±0.12 | 153.33±22.54 | 0.89±0.07 |
|  | T1 | 3.42±0.30 | 155.83±18.53 | 0.85±0.10 |
|  | T2 | 3.94±0.20 | 123.33±13.33 | 1.07±0.07 |
| *Q. falcata* | CK | 3.64±0.50 | 153.39±11.63 | 0.85±0.08 |
|  | T1 | 3.38±0.20 | 83.00±6.33 | 0.91±0.08 |
|  | T2 | 3.70±0.60 | 92.44±15.33 | 1.05±0.12 |
| *Q. texana* | CK | 2.49±0.09 | 245.82±32.50 | 0.84±0.10 |
|  | T1 | 3.42±0.35 | 193.34±44.09 | 0.80±0.03 |
|  | T2 | 2.75±0.35 | 223.33±16.91 | 0.88±0.06 |
| *Q. palustris* | CK | 3.58±0.12 | 216.39±39.60 | 0.77±0.08 |
|  | T1 | 4.33±0.08 | 163.33±15.80 | 0.72±0.15 |
|  | T2 | 4.00±0.16 | 141.90±29.69 | 0.69±0.05 |
| *Q. coccinea* | CK | 3.17±0.12 | 115.78±14.01 | 0.96±0.04 |
|  | T1 | 3.80±0.03 | 89.89±12.93 | 0.89±0.13 |
|  | T2 | 3.89±0.47 | 86.20±7.80 | 0.88±0.09 |
