# Supplementary material for: Morphological Response of Eight Quercus Species to Simulated Wind Load
Source: PLoS One. 2016 Sep 23;11(9):e0163613. doi: 10.1371/journal.pone.0163613 (PMC5035066; doi:10.1371/journal.pone.0163613)
Supplement: S3 Table — SLA, specific leaf area; LDI, leaf dissection index. (DOC) [file pone.0163613.s003.doc]

| species | LDI | SLA | Percentages of root biomass | Stem base diameter |
| --- | --- | --- | --- | --- |
| *Q. acutissima* | 5.34±0.26 | -14.23±1.32 | 15.72±2.16 | -11.79±1.86 |
| *Q. virginiana* | 5.23±0.34 | -15.02±2.13 | 12.31±1.85 | -3.36±0.83 |
| *Q. phellos* | 6.27±0.29 | -15.62±1.67 | 8.08±1.27 | 1.19±0.21 |
| *Q. rubra* | 6.95±0.58 | -16.43±2.01 | 4.18±0.63 | -1.39±0.25 |
| *Q. falcata* | 6.23±0.53 | -11.22±0.33 | 9.14±2.52 | 1.68±0.36 |
| *Q. texana* | 7.8±0.48 | -26.51±2.18 | 3.27±0.18 | 10.24±2.15 |
| *Q. palustris* | 8.05±0.31 | -26.39±1.26 | 0.93±0.04 | 11.63±1.33 |
| *Q. coccinea* | 7.55±0.47 | -20.78±1.13 | 2.66±0.35 | 15.81±0.86 |
